# Propofol sedation reduces contraction and motion of diaphragm in humans: preliminary results

**DOI:** 10.1186/cc14561

**Published:** 2015-03-16

**Authors:** G Ranieri, M Luigi, F Belsito, M Rocco, RA De Blasi

**Affiliations:** 1 Sant'Andrea, Rome, Italy

## Introduction

Among drugs used for sedation, propofol has a primary role [1]. Despite propofol being described to exert a relaxant effect on skeletal muscle, no data showing its action on diaphragm are reported. The aim of this observational study on humans is to apply ultrasound to assess propofol's effect on diaphragmatic contraction and motion during endoscopic procedures.

## Methods

We investigated seven consecutive patients undergoing gastroscopy or colonoscopy in the endoscopy unit of our hospital. Patients received propofol at a dose able to induce and maintain sedation to level 6 of the Ramsay Sedation Scale during the procedure. Measurements were obtained on right side of the thorax in millimeters; diaphragmatic motion (DM) and diaphragmatic motion at maximal inspiration (DM forced) were measured in M-Mode with a 3.5 MHz array convex probe placed on the midclavicular line using the liver acoustic window. Thickness at end inspiration (TEI) and thickness at end expiration (TEE) were measured in M-Mode with a 10 MHz vascular probe. The thickening fraction (TF) was calculated: (TEI - TEE) / TEE [2]. Time points of measurements were taken when the patient arrived in the surgery room (Baseline), 1 minute after level 6 of the Ramsey Sedation Scale was obtained (Sedation) and 5 minutes after the patient had a recovery to level 1 on the Ramsey Sedation Scale (Awakening). Data analyzed are reported in Table 1 and expressed as mean (SD). *ANOVA was used to compare data for repeated measurements. *Post hoc *statistical comparison with Bonferroni's test was used to identify significant variations.

**Table 1:** 

Variable	Baseline	Sedation	Awakening	*P *value
Thickness end inspiration (mm)	3.25 (0.21)	^a^2.66 (0.20)	^b,c^3.22 (0.30)	<0.001^*^
Thickness end expiration (mm)	2.11 (0.20)	^d^2.00 (0.15)	2.07 (0.15)	0.112^*^
Thickening fraction	0.54 (0.07)	^e^0.36 (0.10)	^f,g^0.49 (0.11)	<0.001^*^
Diaphragmatic motion (mm)	18.66 (2.23)	^h^13.27 (4.93)	15.31 (0.40)	0.055
Diaphragmatic motion forced (mm)	54.61 (19.34)	-	56.34 (13.75)	0.298

^a^*P *< 0.001 versus baseline. ^b^*P = *0.043 versus baseline. ^c^*P *< 0.001 versus sedation.

^d^*P = *0.030 versus baseline. ^e^*P =*0.012 versus baseline. ^f^*P =*0.353 versus baseline.

^g^*P = *0.041 versus sedation. ^h^*P = *0.022 versus baseline.

## Results

During propofol administration TEI reduced 19% whereas after awakening it increased 14.5% but did not reach baseline. Conversely TEE did not change during the study. During propofol sedation, TF decreased 34% and returned to baseline after recovery. DM showed 29% reduction during propofol administration whereas the forced diaphragmatic motion tested when patients were conscious (forced DM) did not evidence any change.

## Conclusion

In this observational study, ultrasound assessed that propofol causes a reduction of diaphragmatic contraction and motion during endoscopic procedures.

## References

[B1] Singh H Cochrane Database Syst Rev 2008 4 CD006268 18843709 10.1002/14651858.CD006268.pub2 PMC8988486

[B2] Matamis D Intensive Care Med 2013 39 801 10 10.1007/s00134-013-2823-1 23344830

